# A Cursory Gaze at Solitary Radiopharmaceutical Chelators Incorporating Macrocyclic and Acyclic Moieties

**DOI:** 10.3390/pharmaceutics17121508

**Published:** 2025-11-22

**Authors:** Stephen Ahenkorah, Danni Ramdhani

**Affiliations:** 1 Department of Radiology, The University of Iowa Healthcare, Iowa City, IA 52242, USA; 2 Department of Pharmaceutical Analysis and Medicinal Chemistry, Faculty of Pharmacy, Universitas Padjadjaran, Jl. Ir. Soekarno km 21, Jatinangor 45363, Indonesia; d.ramdhani@unpad.ac.id

**Keywords:** acyclic chelators, macrocyclic chelators, hybrid chelators, radiopharmaceutical, chelating agents, 3p-*C*-NETA, 3p-C-DEPA, chelators

## Abstract

Radiopharmaceutical development usually requires chelators to route radiometals to specific cancer targets. However, there is no universal chelator. To reduce off-target side effects, new chelators are needed as the radiometal toolset grows. DOTA is the most often used chelator for radiometals in radiopharmaceutical development, however DOTA’s extreme conditions make it unsuitable for labeling heat sensitive biological vectors. The ideal chelator should be thermodynamically and kinetically stable, allowing labeling under mild conditions (~37 °C). In recent years, new hybrid chelators with enhanced characteristics have emerged, warranting further investigation for medical applications. This paper aims to discuss some of these promising chelators.

## 1. Introduction

**Figure 1:**
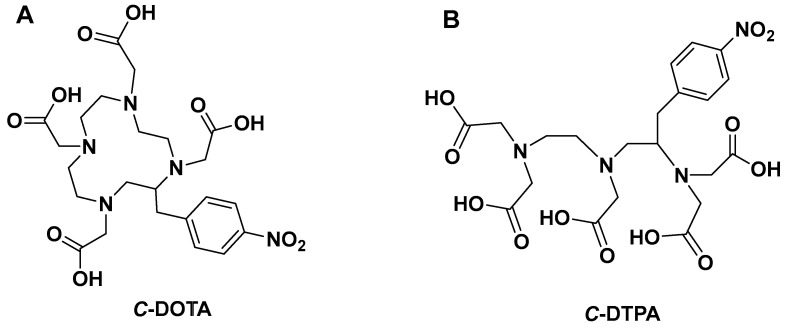
Structures of (**A**) macrocyclic *C*-DOTA and (**B**) acyclic *C*-DTPA.

Most radiopharmaceuticals often used for medical diagnosis and cancer treatment are chelator-based [1,2,3]. The chelating agent binds to the radiometal ion, allowing the biologically targeting vector molecule to release the radioisotope without any radiometal loss from the radiopharmaceutical to produce site-specific targeting for imaging or therapy [3,4,5,6,7]. The chelating agents are, in general, bifunctional chelators (BFCs), which are simply chelating ligands with reactive chemical groups that can be covalently coupled to biologically targeting (bio)molecules (e.g., nucleotides nanoparticles, antibodies, peptides, nanoparticles). It should be highlighted that if these isotopes are to be used safely in medical applications, their unique aqueous coordination chemistry properties must be considered [3,4,6,7]. Currently, chelators utilized in radiopharmaceuticals can be classified into two main structural frameworks: macrocyclic and acyclic [4,8,9,10]. Macrocyclic chelators are ring-shaped while acyclic chelators are open chain (see Figure 1). When designing new chelators, the literature indicates that kinetically macrocyclic chelating agents are more inert than their acyclic counterparts [4,5,11,12]. Macrocyclic chelators require less physical modification during metal ion coordination due to their innately restricted geometries and partially pre-organized metal ion binding sites, which decreases entropic loss. Conversely, acyclic chelators require substantial transformation in geometry and physical orientation in solution to reorientate donor atoms to coordinate with a metal ion, resulting in decrease in entropy [4,5,11]. As a result, macrocyclic chelators have a higher thermodynamic driving force toward complexation than the acyclic counterpart (e.g., Diethylenetriamine pentaacetate; DTPA). The fast dissociation of radiometals from acyclic chelators in vivo is well reported in the literature [3,13].

Coordination kinetics and radiolabeling efficiency are two critical properties that most acyclic chelators excel at, whereas most macrocyclic chelators fail [4]. Acyclic chelators can rapidly complex a radiometal quantitatively under 15 min at 25 °C–40 °C, whereas macrocyclic chelators frequently require high-temperature heating (60 °C–95 °C) for prolonged times (30–90 min) to be quantitative (e.g., 2,2′,2″,2‴-(1,4,7,10-tetraazacyclododecane-1,4,7,10-tetrayl)tetraacetic acid; DOTA) [4,13]. The slow radiolabeling kinetics of macrocyclic agents is well established in the literature [4,13,14]. Fast room-temperature radiolabeling kinetics become critical especially when dealing with BFC-conjugates of thermal-sensitive biomolecules (e.g., antibodies) and short-lived radioisotopes such as scandium-44 (^44^Sc), gallium-68 (^68^Ga), bismuth-212/213 (^212/213^Bi) and copper-62 (^62^Cu) [13].

**Figure 2:**
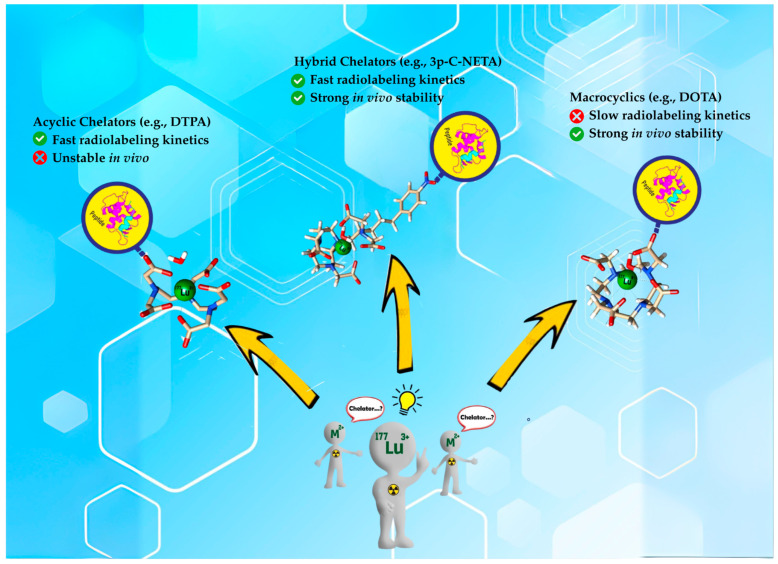
Comparing reaction kinetics and in vivo stability between acyclic, hybrid chelators and macrocyclic.

**Figure 3:**
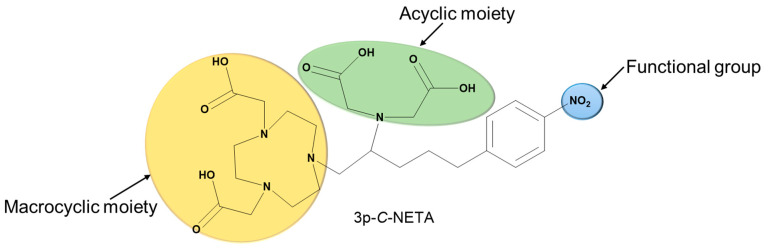
Structure of 3p-*C*-NETA showing the macrocyclic, acyclic and functional groups.

As a result, developing a solitary radiopharmaceutical chelator which incorporates macrocyclic and acyclic frameworks could reap the benefits of these two structurally distinct agents for rapid radiolabeling kinetics and enhanced thermodynamic stability in vivo (Figure 2 depicts the advantages of hybrid chelators over acyclic and macrocyclic chelators). In this review, we will discuss the promising potentials of incorporating macrocyclic and acyclic cores in a single chelator and the future perspective of these interesting agents for radiopharmaceutical development. Figure 3 highlights the structure of a typical hybrid chelator. Yellow indicates the cyclic moiety; green shows the acyclic moiety and blue highlights the functional group.

## 2. 3p-*C*-DEPA

**Figure 4:**
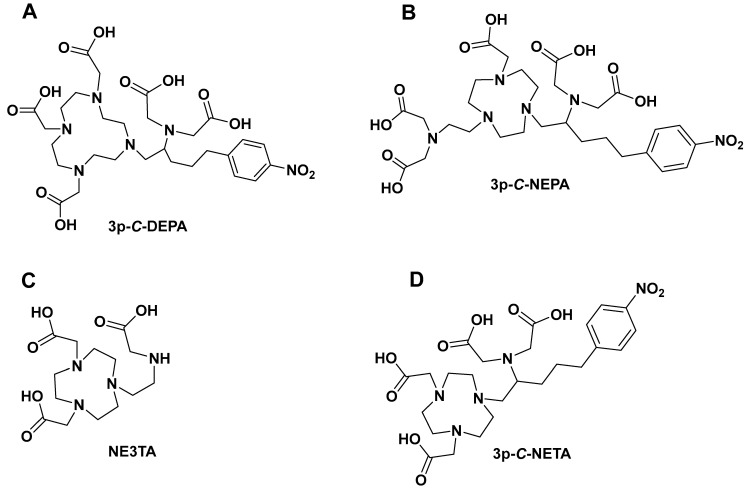
Structures of chelators incorporating macrocyclic and acyclic moieties. (**A**) 3p-*C*-DEPA; (**B**) 3p-*C*-NEPA; (**C**) NE3TA; (**D**) 3p-*C*-NETA.

3p-*C*-DEPA (7-[2-(bis-carboxymethyl-amino)-ethyl]-4,10-bis-carboxymethyl-1,4,7,10-tetraazacyclododec-1-yl-acetic acid (Figure 4) is a chelator which incorporates both an acyclic iminodiacetic acid and macrocyclic DOTA [15,16]. The reason for developing this chelating ligand was to incorporate structural and functionalities of both acyclic and macrocyclic cores, i.e., fast complexation at mild temperatures (25 °C–37 °C) with radioisotopes and high stability in vivo. This hypothesis was confirmed with bismuth-205/206-labeled trastuzumab (^205/206^Bi-trastuzumab). ^205/206^Bi labeling of 3p-*C*-DEPA-trastuzumab conjugate was extremely rapid (1 min, RCY > 93%, 0.25 M NH_4_OAc, pH 5.5) at 25 °C. The complex remained intact in human serum (37 °C) up to 72 h with no measurable dissociated activity [15]. Good complexation kinetics (0.25 M NH_4_OAc, pH 4.0, 45 °C, 30 min) and excellent in vitro human serum stability (37 °C, >98% intact up to 48 h) of the DEPA chelator as such with ^177^Lu has also been reported [15,16].

**Figure 5:**
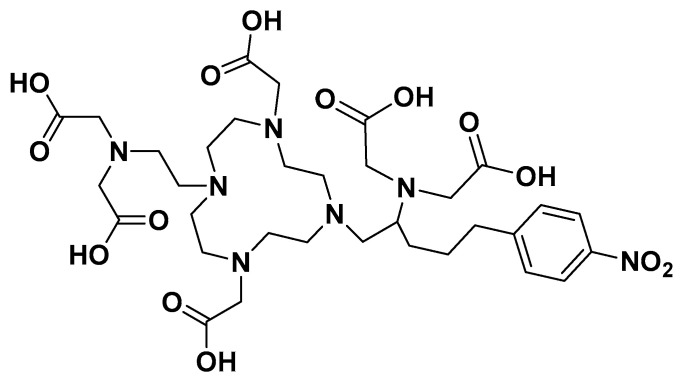
The structures of dodecadentate analog of DEPA.

Kelly et al. has also performed studies comparing the stability and complexation kinetics of DOTA, 3p-*C*-DEPA (Figure 4) and a derivatized DEPA (dodecadentate analog, Figure 5) conjugated to PSMA with ^68^Ga, indium-111 (^111^In), lutetium-177 (^177^Lu) and actinium-225 (^225^Ac). The goal of this study was to determine how metal size and coordination number interact with macrocyclic chelators of different denticity. The three PSMA-conjugated chelating ligands demonstrated similar complexation to ^68^Ga; however, even though the DEPA derivatives provided RCY > 20%, DOTA was the poorest with RCY < 10% at 25 °C (30 min). A similar trend was observed for ^111^In as RCY > 88% by the DEPA derivatives, while DOTA yielded RCY < 40% at 25 °C. DOTA and DEPA dodecadentate analog were nearly quantitative for ^177^Lu 95 °C. Chelation kinetics was in favor of the DEPA derivative according to the authors. For example, at 5 µg of each ligand, RCY > 66% and RCY > 92% were observed for DOTA and DEPA derivative, respectively, at 1 min (95 °C). At 25 °C, the authors observed a striking difference in reaction kinetics between DOTA and DEPA derivatives. DEPA dodecadentate displayed time- and mass-dependent complexation to ^177^Lu, i.e., RCY reaching approximately 44% (5 µg), 45% (15.5 µg) and 65% (25 µg) at 30 min. Contrary to this observation DOTA produced RCY < 10% at the same reaction conditions, highlighting the fast kinetics of chelators incorporating the core of acyclic and macrocyclic chelators. The authors studied radiolabeling of ^225^Ac at 95 °C. DEPA dodecadentate analog produced an excellent RCY of approximately 95% at all masses tested at 15 min. The researchers reported that reaction kinetics were mass dependent. DOTA demonstrated slower reaction kinetics initially; however, RCY of 93% was reached at 15 min for all masses tested. This result was not surprising as DOTA is known to undergo decent reaction kinetics at elevated temperatures. Future reaction at 25 °C is critical to determine how the DEPA derivatives will perform. The rapid complexation of DEPA compared to DOTA may be due to the additional donor atoms on the acyclic core. In general, more donor atoms allow for the formation of more chelate rings, which is desired due to the entropic benefit. Furthermore, the probability that the complexed metal will reassociate rather than dissociate is extremely high. This is because while one donor atom dissociates, other atoms remain connected to the metal, providing the broken bond with a better chance of reforming again. Based on this data, the structure of the DEPA derivatives and the relatively large ionic radius of ^225^Ac, we can speculate that these chelating ligands might be interesting for the alpha emitter ^225^Ac for targeted alpha therapy [17].

## 3. 3p-*C*-NEPA

A scorpion-like chelator, 2,2′-((2-(4-(2-(bis(carboxymethyl)amino)-5-(4-nitrophenyl)pentyl)-7-(carboxymethyl)-1,4,7-triazonan-1-yl)ethyl)azanediyl)diacetic acid (3p-C-NEPA, Figure 4), which has ten possible donor atoms and acyclic arms, is reported for fast and complete saturation of the coordination sphere of a metal ion with a large ionic radius by Sun et al. 3p-*C*-NEPA rapidly formed a complexation to ^90^Y or ^177^Lu (1 min, RCY > 97%, pH 5.5, 0.25 M NH_4_OAc, 25 °C). ^177^Lu-3p-*C*-NEPA and ^90^Y-3p-*C*-NEPA were stable in human serum for up to 14 days (>97% intact radiocomplex) [18]. In vivo biodistribution data of ^177^Lu-3p-*C*-NEPA showed an excellent biodistribution profile, with rapid blood clearance and low uptake in normal tissues. Polydentate ligands can form multiple bonds concurrently, resulting in chelate rings that improve thermodynamic stability and increasing donor atoms in polydentate ligands can increase chelation effect. This chelation effect minimizes the possibility of dissociation from the metal, making polydentate ligands more efficient in stabilizing metal ions. Based on this assumption, we can speculate that the presence of ten donor sites (N and O) on 3p-*C*-NEPA may contribute to its fast radiolabeling kinetics as it can occupy ten sites on the metal core, potentially resulting in rapid complexation and strong in vivo stability. More studies such as radiolabeling with different short-lived radioisotopes will be critical for the future translation of this agent into the clinic.

## 4. NE3TA Derivatives

### 4.1. C-NE3TA and N-NE3TA

(7-[2-[carboxymethyl)amino]ethyl]-1,4,7-triazacyclononane-1,4-diacetic acid; NE3TA)-based heptadentate chelates containing both the macrocyclic TACN backbone and a pendant arm has been reported [19]. The acyclic binding moiety attached to the TACN core was proposed to improve complexation kinetics by rapidly binding to ^64^Cu, which can lead to solvation by the TACN donors resulting in a stable metal–chelator complexation. Another group have demonstrated that the bifunctional chelating agents *C*-NE3TA (7-[2-[carboxymethyl)amino]-3-(4-nitrophenyl)propyl]-1,4,7-triazacyclononane-1,4-diacetic acid) and *N*-NE3TA (7-[2-[(carboxymethyl)amino]-2-(4-nitrophenyl)methyl]-1,4,7-triazacyclononane-1,4-diacetic acid) were stable in human serum (>91% intact) after 48 h [20]. ^64^Cu-labeled *N*-NE3TA and *C*-NE3TA also showed rapid clearance in vivo. To explore NE3TA further, the authors evaluated the bifunctional chelator 3p-*C*-NE3TA (derivatized NE3TA) for radiocomplexation kinetics, in vitro and in vivo stability with ^64^Cu. In this study, using DOTA as a reference, the authors reported 3p-*C*-NE3TA formed a rapid complexation with ^64^Cu at 25 °C (1 h, pH 5.5, 0.25M NH_4_OAc); however, under the same conditions, DOTA required 5 h to form complexation with ^64^Cu, hence, the temperature was increased to 90 °C (1 h) in subsequent complexation reactions for DOTA.

### 4.2. NE3TA-PY

**Figure 6:**
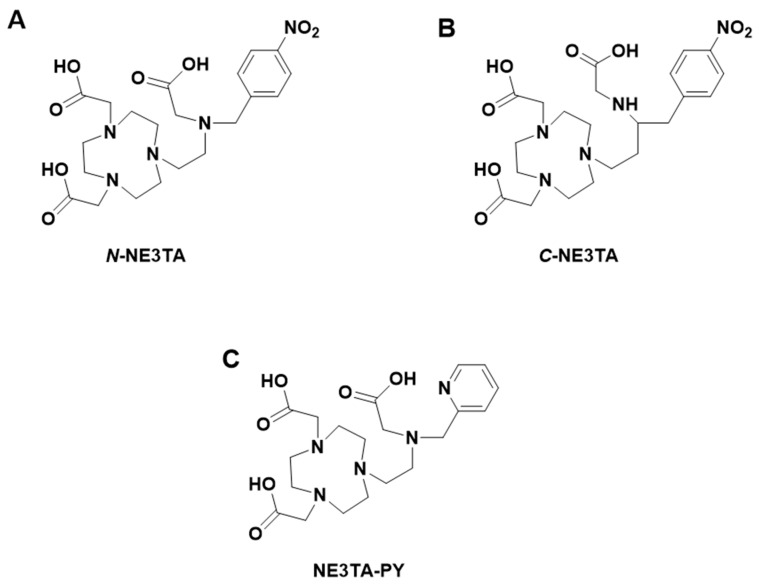
Structures of (**A**) *N*-NE3TA; (**B**) *C*-NE3TA; (**C**) *C*-NE3TA-PY.

NE3TA-PY (7-[2-[(carboxymethyl)(2-pyridylmethyl)amino]ethyl]-1,4,7-triazacyclononane-1,4-diacetic acid, Figure 6), a derivatized version of NE3TA, has also been studied with ^177^Lu [21]. This chelator was developed to improve the complexation kinetics of NE3TA radiocomplexes. NE3TA-PY rapidly and nearly quantitatively bound to ^177^Lu under 1 min (RCY = ~93%, pH 7.0, 0.25 M NH_4_OAc, 25 °C) [21]. Also, ^177^Lu-NE3TA-PY was stable in human serum for up to 7 days without a detectable dissociation in the serum media. The authors explained that the observed rapid complexation of NE3TA-PY with radionuclides under neutral conditions (pH 7, 25 °C) could be due to the enhanced ligating capability of the basic nitrogen atom in the pyridyl ring. *p*-SCN-PhPr-NE3TA, which is a functionalized version of NE3TA, has also been developed for positron emission tomography (PET) imaging [22]. *p*-SCN-PhPr-NE3TA demonstrated the ability to form stable Cu(II) complexes and excellent chelating selectivity. Copper-64 (^64^Cu)-labeled *p*-SCN-PhPr-NE3TA under mild conditions provided high specific activity (0.1 M NH_4_OAc, pH 4.0, RCY = 90%, 37 °C, 30 min) [22]. The radiocomplex demonstrated excellent in vitro stability in human serum (37 °C, >98% intact tracer, 24 h). The authors added that *p*-SCN-PhPr-NE3TA could be a promising chelating agent for biomolecule-based radiotracers [22].

## 5. 3p-*C*-NETA

Another typical example of these macrocyclic- and acyclic-infused chelators is 3p-*C*-NETA (4-[2-(bis-carboxy-mehylamino)-5-(4-nitrophenyl)-entyl]-7-carboxymethyl-[1,4,6]tri-azonan-1-yl} acetic acid (Figure 3). 3p-*C*-NETA possess both a parent macrocyclic NODA (1,4,7-triazacyclononane-*N*,*N*′-diacetic acid) backbone and a flexible acyclic tridentate pendant arm. Chong et al. demonstrated the promising characteristics of 3p-*C*-NETA regarding their rapid complexation and stability with yttrium-90 (^90^Y) and ^177^Lu [23,24,25]. The complexation of 3p-*C*-NETA with ^90^Y and ^177^Lu (>95%) occurred under 5 min. This interesting outcome triggered Kang et al. to evaluate ^90^Y- and ^177^Lu-3p-*C*-NETA- trastuzumab stability in vitro and in vivo pharmacokinetic properties in tumor-bearing mice. Rapid radiolabeling kinetics and stability studies of ^205/206^Bi-complex have also been reported [24]. This promising data sparked the interest of Ahenkorah et al. in assessing the radiolabeling of 3p-*C*-NETA with different imaging and therapeutic radionuclides (^225^Ac, ^213^Bi, ^177^Lu, ^67^Cu and ^161^Tb) and imaging radionuclides ([^18^F]AlF and ^68^Ga). The in vitro stability of the resulting radiocomplexes was evaluated. 3p-*C*-NETA conjugated to an octreotate with Al^18^F was also evaluated. 3p-*C*-NETA was demonstrated to be a versatile chelating agent for both imaging applications ([^18^F]AlF) and therapy (^213^Bi, ^177^Lu, ^161^Tb), demonstrating the need to further evaluate this promising chelator for future translation into the clinic [14,26]. Refer to Ahenkorah et al. (2022) [14] for more details on this study. The significant chelate effect observed in all macrocyclic polydentate systems can be attributed to their entropic advantage; the more donor atoms in a single chelate, the more stable the chelate–metal complex. Hybrid chelators, such as 3p-*C*-NETA, produce exceptionally stable chelator–metal complexes, which are clearly owing to more than just the chelate effect. We propose that this improved stability is attributable to both the donor atoms’ pre-organization on the acyclic arms and the entropic advantage. Another intriguing contributing factor could be the “Hard-Soft Acid-Base” theory (HSAB). This hypothesis divides ligands (bases) and metals (acids) into distinct groups based on their “hard” and “soft” properties. To be considered a hard acid, a metal must meet the following conditions: (1) small ionic size and high charge; (2) low polarizability; and (3) a strong affinity for hard bases. Radiolabeling experiments with 3p-*C*-NETA showed a strong match with [^18^F]AlF, ^213^Bi, ^177^Lu and ^161^Tb. Applying the HSAB theory, we can conclude that 3p-*C*-NETA prefers hard acids ([^18^F]AlF, ^177^Lu, ^161^Tb); however, its ability to undergo rapid complexation and strong stability with ^213^Bi, which is a borderline between hard and soft acids, demonstrates the versatility of this promising chelating agent, and it may be a safe alternative to DOTA.

## 6. Discussions and Future Perspective

The scale of radionuclide production has increased dramatically in recent years as radiopharmacy has gained popularity and medical cyclotrons have become more widely available [27,28]. These developments have inspired an increase in research into radiometals, which were previously unavailable but had promising features for radiopharmaceutical uses. This is demonstrated by the large number of promising metal-based radiopharmaceuticals approved by the FDA/EMA, which has substantially enhanced confidence in the radiopharmaceutical sector. This development has driven the search for matched chelating agents capable of stably complexing these radiometals. The chelating ligand must form a highly stable, ring-like complex with the target metal ion and exhibit great selectivity for the matching radiometal. Additionally, this agent must include at least two atoms (e.g., oxygen, nitrogen or sulfur) with accessible electron pairs capable of forming coordinated bonds with the metal ion at the same time [4]. The resulting radiometal-chelate complex must be thermodynamically stable, with a high formation constant to keep the metal ion strongly attached and effectively isolated from other biological reactions. Finally, to enhance binding affinity, the chelating agent’s donor atoms should be “hard” or “soft” in character, matching the “hard” or “soft” nature of the target metal ion, according to the HSAB principle. DOTA and NOTA remain the most widely used chelating agents in the field of radiopharmacy. NOTA, for example, forms a highly stable complex with Ga(III) at room temperature, making it a suitable option for thermal-sensitive biomolecules. However, the structure of NOTA (smaller in size) limits its usage with a wide range of radioisotopes such as ^177^Lu and ^161^Tb, making it unpopular for clinical applications. DOTA, as mentioned elsewhere, is the preferred chelating agent for radiopharmaceutical production. However, kinetic measurements indicate that the first step in the formation of radiocomplexes with macrocyclic ligands involves the rapid formation of a mono- or diprotonated intermediate, with the base-assisted reorganization of this intermediate determining the rate. These protonated intermediates slow down the reaction kinetics and have been proven to exist by NMR spectroscopy in some cases [4]. The slow formation kinetics of macrocyclic-based ligands could be a major drawback in the long term, limiting the practical application of chelator-based radiopharmaceuticals. To overcome this kinetic energy barrier, high temperatures are frequently used during the complexation reaction to accelerate the chelation process. Several chelating ligands have been developed to improve reaction kinetics and stability. For example, DOTAGA, a derivatized form of DOTA, is a promising chelator that offers a higher labeling efficiency compared to DOTA. However, its higher kidney uptake and a negative charge have stalled its translation to the clinic [4]. Hence, further research to fully understand this chelating agent is crucial. Macropa, a macrocyclic chelator with two pendant picolinic side arms, has also made its way into the field of radiopharmaceutical development, particularly for the complexation of ^225^Ac. Macropa forms an extremely stable complex with the trivalent cation ^225^Ac; however, given the size of macropa and the limited stability data, particularly for radiopharmaceuticals in vivo applications, further investigation into other novel chelating ligands is necessary [29]. These limitations have necessitated the need for alternative chelating ligands that could provide enhanced reaction kinetics and stability at ambient temperature.

Additionally, in radiopharmaceutical development, the pharmacokinetics of the imaging and therapeutic agent must be nearly equal or very comparable to have a true theranostic pair that enables precise, customized dosimetry estimation for radionuclide therapy. The utilization of a single vector molecule to produce both imaging and therapeutic agent with very similar biodistribution profile in the body may therefore be advantageous. This can cut production cost and reduce the production time for the precursor. Another advantage of using the same precursor agent to develop imaging and therapeutic radiotracer is the added benefit of requiring only a single GMP-grade molecule to be developed and produced, which reduces cost drastically and manufacturing time. Hybrid chelators such as 3p-*C*-NETA have demonstrated to be capable of singularly providing the combined advantages of macrocyclic and acyclic chelators. It is a versatile chelator that can complex a wide range of diagnostic and therapeutic radioisotopes. Also, synthesis of these ligands is quite straightforward, based on the literature, making scalability possible. Hence, we hypothesize that single chelators with acyclic and macrocyclic moieties can (1) offer versatility in the production of theranostic pairs; (2) achieve effective complexation with radioisotopes at room temperature, which will be crucial for heat-sensitive biomolecules and short-lived radionuclides; (3) preserve the pharmacokinetic characteristics of the biological targeting molecules in vivo; and (4) reduce the production time of radiopharmaceuticals, since a single chelator will be used for both diagnostic and therapeutic applications.

## 7. Conclusions

Advances in radiopharmacy necessitate a deeper understanding of chelator-based radiopharmaceuticals, particularly those containing the cores of acyclic and macrocyclic chelators. As a result, this review outlined the currently available hybrid chelators that incorporate these cores for radiopharmaceuticals. Radiolabeling kinetics (e.g., macrocyclic vs. acyclic) must be considered to maximize the in vivo behavior of a radiopharmaceutical, which necessitates the exploration of a wide range of these hybrid chelators. We anticipate further research into these chelators in other applications, with the potential to become the new ‘gold standard’.

## Data Availability

Data sharing is not applicable. No new data were created or analyzed in this study.
